# Development of a School-Based Intervention to Increase Physical Activity Levels Among Chinese Children: A Systematic Iterative Process Based on Behavior Change Wheel and Theoretical Domains Framework

**DOI:** 10.3389/fpubh.2021.610245

**Published:** 2021-04-27

**Authors:** Haiquan Wang, Holly Blake, Kaushik Chattopadhyay

**Affiliations:** ^1^Division of Epidemiology and Public Health, School of Medicine, University of Nottingham, Nottingham, United Kingdom; ^2^The Nottingham Centre for Evidence-Based Healthcare: A Joanna Briggs Institute Centre of Excellence, Nottingham, United Kingdom; ^3^School of Health Sciences, Faculty of Medicine and Health Sciences, University of Nottingham, Nottingham, United Kingdom; ^4^National Institute for Health Research Nottingham Biomedical Research Centre, Nottingham, United Kingdom

**Keywords:** China, exercise, sport, child, systematic process, intervention development

## Abstract

Regular physical activity has a range of benefits for children's health, academic achievement, and behavioral development, yet they face barriers to participation. The aim of the study was to systematically develop an intervention for improving Chinese children's physical activity participation, using the Behavior Change Wheel (BCW) and Theoretical Domains Framework (TDF). The BCW and TDF were used to (i) understand the behavior (through literature review), (ii) identify intervention options (through the TDF-intervention function mapping table), (iii) select content and implementation options [through behavior change technique (BCT) taxonomy and literature review], and (iv) finalize the intervention content (through expert consultation, patient and public involvement and engagement, and piloting). A systematic iterative process was followed to design the intervention by following the steps recommended by the BCW. This systematic process identified 10 relevant TDF domains to encourage engagement in physical activity among Chinese children: knowledge, memory, attention and decision processes, social influences, environmental context and resources, beliefs about capabilities, beliefs about consequences, social/professional role and identity, emotions, and physical skills. It resulted in the selection of seven intervention functions (education, persuasion, environmental restricting, modeling, enablement, training, and incentivization) and 21 BCTs in the program, delivered over a period of 16 weeks. The BCW and TDF allowed an in-depth consideration of the physical activity behavior among Chinese children and provided a systematic framework for developing the intervention. A feasibility study is now being undertaken to determine its acceptability and utility.

## Background

Health benefits of physical activity among children are vast (1, 2). Regular participation in physical activity can improve children's overall health (e.g., cardiovascular health, mental health, musculoskeletal health) and can contribute to their social well-being (3). Evidence shows low physical activity levels among children, and physical inactivity continues to be a major public health problem globally (4). The World Health Organization's (WHO) physical activity guideline recommends a minimum of 60 min of moderate-to-vigorous physical activity (MVPA) per day among children aged 5–17 years, including vigorous activities and activities that strengthen muscles and bones at least 3 days per week (1). WHO also recommends that these children should limit the amount of time spent on being sedentary and recreational screen time in particular (1). In China, however, only 34% of school-aged children have met the recommended 60 min of MVPA per day, and only 9% of children have spent at least 3 days on vigorous activities per week (5, 6). Around 35% of children have failed to adhere to the recommendations on sedentary and recreational screen time (5, 6). Boys are more likely to meet the MVPA recommendations compared to girls. However, boys are less likely to meet the sedentary and recreational screen recommendations compared to girls (7). A range of factors may influence a child's physical activity, including (i) personal (relating to physical, emotional, or mood-associated factors among children), (ii) socio-cultural (relating to people with whom the child would come in contact with, such as parents/guardians and teachers), (iii) environmental (relating to structural elements such as facilities and transport), and (iv) policy- and program-related (relating to programs, organizations, and staff) factors (8–14). MVPA starts to decline among children at around 13 years of age irrespective of their residence (i.e., urban or rural) (5, 15, 16). These factors are interconnected and important at different stages across the lifespan, with certain aspects being more influential at different points across the life course. Approximately 25% of Chinese children have spent over 30 min on MVPA in primary school (aged 7–12 years) whilst only 15% and 10% of junior middle children (aged 13–15 years) and junior high children (aged 16–18 years), respectively (10). This is consistent with several national and international surveys that have reported children's physical activity starts to decline at 10–12 years of age (17, 18). In other words, it can be beneficial to target health behaviors (including physical activity) at this transition period as children approach adolescence.

In China, children are under huge academic pressure as a result of the national exam-oriented education system. Over one-third of these children report psychosomatic symptoms at least once a week, and around 76% of them report being in a bad mood because of academic pressure and high parental expectation (19). This pressure increases from junior middle school to junior high school (20). On weekdays, the school hours last for ~9 h for Chinese children in primary school, which is more intensive than in the United States (US) and United Kingdom (UK) (21–23). In schools, health (physical) education and structured exercise programs are available and delivered to the children orally and/or in written format. Structured exercise sessions are run to achieve the recommended intensity and duration of physical activity. However, evidence suggests that these programs either do not intervene on the children's intrinsic determinations to physical activity or physical activity that occurs after school (e.g., weekends and holidays) (24). At the administrative level, a “shrinkage” of physical activity time for children has been identified due to the low enforcement of physical activity policy and unorganized school support (24, 25). Moreover, there has been a consistent decline in physical activity time among Chinese children over the past two decades (26). Health education sessions are often replaced by other elements of the academic curriculum due to bad weather or during periods when children are due to undergo academic assessments (24). Health education has not historically been valued within the curriculum because it is not a mandatory module listed on the national university entrance exam (24, 27). In addition, the number of health education sessions set up for children reduces as they move into higher classes (27). In turn, the organization of health education becomes relatively insignificant in Chinese schools, which fails to provide a supportive physical activity environment and appropriate physical activity education or values for the children, parents, and teachers. Moreover, the development process of these programs remains questionable, and most of these programs are not based on behavior change theories (28–30). For instance, a systematic review of the effectiveness of physical activity programs has suggested that the programs in China delimit the rigorous process of development and evaluation (30–32). Around 80% of these physical activity programs are found to be of poor quality. Furthermore, the program designs lack a theoretical basis for the analysis regarding potential drivers of target behaviors (30–32). The quality of interventions could be improved through consulting experts and by having patient and public involvement and engagement (PPIE) during intervention development, dissemination, and implementation; however, previous studies have often neglected or not reported these steps in the intervention development (33). This could be due to the associated costs of PPIE, short timescales for delivery of interventions, not understanding the importance of PPIE steps, or having a tight word limit in peer-reviewed publications.

Theory-based interventions are more likely to succeed and could help to elucidate why and how the intervention components may contribute to the overall effectiveness (34). In contrast, non-theory based interventions are less likely to be successful as they may fail to translate the existing scientific evidence into knowledge and practice and neglect the potential explanatory underpinnings of the target behaviors or problems (35, 36). As such, there is a need to develop theory-based interventions. The Behavior Change Wheel (BCW) and Theoretical Domains Framework (TDF) are models that could provide a systematic and comprehensive assessment of the factors that are likely to influence behaviors (37, 38). Specifically, the BCW is a synthesis of 19 frameworks of behavior change and is based on a Capability Opportunity Motivation Behavior (COM-B) model, which is fundamental to the identification of changes needed for desired behavior and selection of associated intervention functions and behavior change techniques (BCTs) (34, 37). Theoretically, the COM-B model assumes that interactions between an individual's capability, opportunity, and motivation can explain why a particular behavior is or is not performed (34). Each COM-B component can be further subdivided into two categories. Capacity can be physical (e.g., physical skills, strength) or psychological (e.g., knowledge, psychological skills) capacity to engage in the activity concerned. Opportunity can be physical (e.g., time, resource) or social (e.g., cultural norms, interpersonal influences) factors that lie outside the individual that prompt the behavior. Motivation can be reflective (e.g., self-conscious intentions, plans) or automatic (e.g., emotional reactions, desires) brain processes that energize and direct the behavior. Altogether, the COM-B model provides a systematic and comprehensive way to guide the behavior change analysis to bring about the desired behavioral change. The TDF is an elaboration of the COM-B that consists of 14 theoretical domains and can be used for a detailed analysis of potential barriers and facilitators to be targeted in an intervention (38). In total, a set of 14 domains covering the main factors influencing a practitioner's clinical behavior and behavioral changes were identified, including (i) knowledge, (ii) skills, (iii) social/professional role and identity, (iv) beliefs about capabilities, (v) optimism, (vi) beliefs about consequences, (vii) reinforcement, (viii) intentions, (ix) motivation and goals, (x) memory, attention, and decision processes, (xi) environmental context and resources, (xii) social influences, (xiii) emotion, and (xiv) behavioral regulation (38). Overall, these 14 domains provide an extensive framework to prompt the consideration of good coverage of influencers to behavioral change and therefore improves the intervention implementation. The BCW has been successfully used to inform the design of many interventions that target a variety of health-related issues, such as stroke rehabilitation, auditory rehabilitation, and cancer symptom awareness (39–41). Moreover, it has been successfully used to develop physical activity interventions in both children and adults in high-income countries (42, 43). However, there remains a dearth of research using the BCW and TDF to develop a physical activity intervention in an upper middle-income country like China (44, 45). Physical activity interventions are likely to be complex interventions as several components interact with those who deliver and those who receive the intervention. It is also needed to take account of the role of parents and the environment for physical activity. Developing an intervention that is aimed at increasing children's physical activity level has much to do with behavioral change (46). This is because intervening through the promotion of physical activity is a long-term behavior change process that should include both behavior initiation and maintenance. However, previous studies have paid little attention to behavioral changes of children and people who have responsibility for them (i.e., parents, guardians, or teachers) in the development and implementation of complex interventions (30–32). Similarly, previous studies have understated the role of policy- and program-related factors (i.e., sports programs, organizations, and government departments) in the development process. Interventions aimed at changing Chinese children's behavior have generally proven problematic in demonstrating efficacy, possibly because of a lack of or inadequate theoretical foundation in the development phase (30). Furthermore, interventions have been criticized for lacking a theoretical rationale and detailed reporting, thus complicating both the development and the possibility of replicating or improving interventions (34).

The application of the BCW varies from one context or behavior to another. For instance, the barriers and facilitators to children's physical activity differ in different cultural contexts. As such, a theory-based physical activity intervention is needed in China. Thus, the aim of the study was to use BCW and TDF for designing the components of an intervention to improve Chinese children's (aged 10–12 years) physical activity. Specifically, this is the first stage of the UK Medical Research Council's (MRC) widely used framework for developing and evaluating complex interventions, which describes four phases: intervention development, feasibility/piloting, evaluation, and implementation (46). The specific objectives were to (i) identify barriers and facilitators to physical activity in ethnic Chinese children and determine which barriers and facilitators need to be addressed in different settings (e.g., home, school, community), (ii) identify the type of intervention functions required to bring about the change, (iii) identify specific BCTs aligned to the purpose of physical activity promotion, and (iv) develop the intervention content and structure for the physical activity program.

## Methods

**Figure 1:**
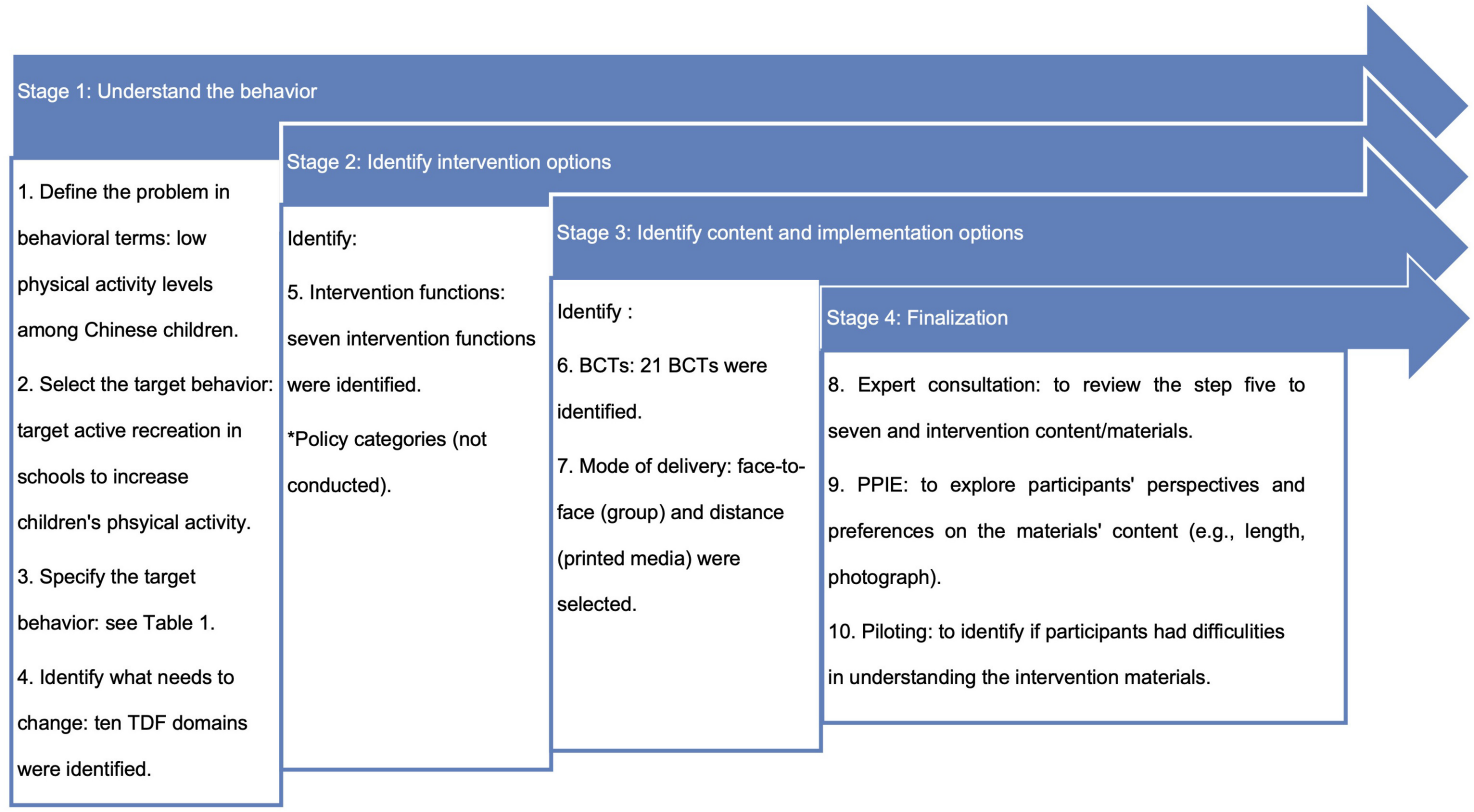
The 10 steps of the intervention development.

BCW and TDF intervention development steps were followed, and this was an iterative process (e.g., going back to the previous step and making changes based on the feedback) (37). In the BCW, the intervention design has eight steps: (i) defining the problem in behavioral terms, (ii) selecting the target behavior, (iii) specifying the target behavior, (iv) identifying what needs to change, (v) identifying intervention functions, (vi) identifying policy categories, (vii) identifying BCTs, and (viii) identifying the mode of delivery (37). We broadly followed these steps (except for identifying policy categories) along with the process of expert consultation, PPIE, and piloting in developing the physical activity intervention (see Figure 1). Reporting of the developed intervention is in accordance with The Template for Intervention Description and Replication (TIDieR) (see Table 1) (47).

**Table 1:** Description of the finalized intervention and implementation strategy using the TIDieR checklist.

**Item number**	**Item**	
	**Brief name**	
1.	Provide the name or a phrase that describes the intervention.	Development of a behavior change intervention to increase physical activity among children aged 10–12 years in China.
	**Why**	
2.	Describe any rationale, theory, or goal of the elements essential to the intervention.	This study is aimed to develop a school-based behavior change intervention to increase physical activity among children aged 10–12 years in China. The intervention was systematically developed based on the behavior change wheel and theoretical domains framework.
	**What**	
3.	Materials: describe any physical or informational materials used in the intervention, including those provided to participants or used in intervention delivery or training of intervention providers. Provide information on where the materials can be accessed (e.g., online Appendix, URL).	This intervention involves three levels, including (i) environmental support in school, (ii) health education for children, and (iii) family involvement. Materials used for each level are: (i) School environmental support: (i) Physical activity poster (ii) Sports equipment for children (ii) Health education for children: (i) Slides (ii) An activity diary (iii) Family involvement: (i) Slides (ii) A physical activity booklet (iii) Physical activity expert clipsTraining of intervention providers includes training in measurement of body weight, height, and waist circumference.Information on the development of intervention materials and the source of materials are mentioned in the “Result section” (steps 8, 9, 10).
4.	Procedures: describe each of the procedures, activities, and/or processes used in the intervention, including any enabling or support activities.	This intervention involved three levels, including (i) environmental support in school, (ii) health education for children, and (iii) family involvement. (i) School environmental support The environmental support in school is provided to children by the physical education teachers and the researcher (HW) from the beginning of the intervention until the end. The support consists of (i) verbal encouragement for doing physical activity at the class break, (ii) provision of physical activity posters in the classroom, and (iii) provision of sports equipment for children to use during the class break. (ii) Health education for children Health education for children is a multi-faceted intervention and aims to increase children's knowledge, motivations for physical activity and help them make their own physical activity plans. The multi-faceted strategy consists of (i) a general introduction to physical activity (definition, positives, negatives of physical inactivity) using slides, (ii) discussion about what sort of things motivate them to do physical activity/what stops them doing it and the previous successful physical activity experience, (iii) tips for doing physical activity (intense, duration, safety) and motivation, (iv) encouragement for children to make physical activity plans to be active at school and home gradually (whenever the goal or plan is achieved, verbal congratulations or non-verbal rewards will be offered to them), (v) encouragement for children to make physical activity poster and organization of presentation, (vi) provision of follow-up educational material (e.g., activity diary) for each child, and (vii) encouragement for children to set up activity goals and record their daily physical activity (steps) using the activity diary. (iii) Family involvement Family involvement is provided to parents and it aims at encouraging parents to support their children in doing physical activity. The strategy consists of (i) a session of health education for parents in terms of physical activity knowledge and ways to promote physical activity among children (using slides), (ii) utilization of video clips as the demonstrations (from experts), (iii) encouragement for parents to discuss the barriers that hinder children's physical activity as well as the facilitators that can enable physical activity among children, (iv) encouragement for parents to support children in doing physical activity (e.g., making action plans) and (v) provision of follow-up educational material (e.g., a booklet) for each parent to reflect on the knowledge of physical activity.
	**Who provided**	
5.	For each category of intervention provider (e.g., psychologist, nursing assistant), describe their expertise, background, and any specific training given.	(i) School environmental support The school environmental support is provided by physical education teachers and HW. The physical education teachers are eligible if he/she is the teacher of the children in our intervention. Sports equipment is purchased and provided by HW. (ii) Health education for children Health education for children is delivered by HW with expertise in health behavior change, physical activity in conjunction with the physical education teacher with expertise in education and safeguarding. (iii) Family involvement Health education for parents is delivered by HW with expertise in health behavior change, physical activity in conjunction with the physical education teacher with expertise in education and safeguarding.
	**How**	
6.	Describe the modes of delivery (e.g., face-to-face or by some other mechanism, such as internet or telephone) of the intervention and whether it was provided individually or in a group.	(i) School environmental support The school environmental support (i.e., a poster and sports equipment) is delivered to the children's class at the start of the intervention by HW. (ii) Health education for children Health education for children (including discussion, plan-making, poster-making, and presentation) is delivered to children in four face to face group sessions by HW (with the presence of the physical education teacher) using Powerpoint slides. The activity diary is used as an educational material delivered to children individually in hardcopy format at the start of this intervention. (iii) Family involvement Health education for parents (including discussion) is delivered to parents in an online session by HW (with the presence of the physical education teacher) using the slides. The physical activity booklet is used as an educational material delivered to parents individually in hardcopy format at the start of this intervention.
	**Where**	
7.	Describe the type(s) of location(s) where the intervention occurred, including any necessary infrastructure or relevant features.	(i) School environmental support The School environmental support (i.e., a poster and sports equipment) is delivered at the school (intervention group). (ii) Health education for children Health education for children (including discussion, plan-making, poster-making, and presentation) is delivered at the school (intervention group). (iii) Family involvement Health education session for parents (including discussion) is delivered remotely through an online platform (intervention group).
	**When and how much**	
8.	Describe the number of times the intervention was delivered and over what period of time including the number of sessions, their schedule, and their duration, intensity, or dose.	(i) School environmental support The School environmental support is delivered at the beginning of the intervention and it lasts for 16 weeks. (ii) Health education for children Health education for children is delivered four times over 45-min sessions at week 1, 5, 9, and 12. Ongoing supports are available for children from the team subsequently. (iii) Family involvement Health education session for parents is delivered once over a 45-min session, at week 1. Ongoing supports are available for parents from the team subsequently.
	**Tailoring**	
9.	If the intervention was planned to be personalized, titrated, or adapted, then describe what, why, when, and how.	(i) School environmental support The School environmental support is standardized through the use of a printed poster and purchased sports equipment. It is also acknowledged that additional unforeseen questions from children may arise which HW may have to deal with on a case-to-case basis. (ii) Health education for children Health education for children is standardized using slides and printed activity diary. These materials are developed by the study team with input from the six physical activity and behavior change experts. (iii) Family involvement Health education for parents is standardized using slides and printed physical activity booklet. These materials are developed by the study team with input from the six physical activity and behavior change experts.
	**Modifications**	
10.	If the intervention was modified during the course of the study, describe the changes (what, why, when, and how).	N/A
	**How well**	
11.	Planned: if intervention adherence or fidelity was assessed, describe how and by whom, and if any strategies were used to maintain or improve fidelity, describe them.	A feasibility study is registered with the Chinese Clinical Trial Registry (ChiCTR1900026865) and is being conducted to test the intervention procedures for its acceptability, such as estimating recruitment and retention, determining the sample size, and optimizing the intervention. Specifically, a detailed protocol is developed which included the selection of appropriate data collection tools to measure the change in children's physical activity. Adherence and fidelity are measured by the registry record and the practitioner questionnaire.
12.	Actual: if intervention adherence or fidelity was assessed, describe the extent to which the intervention was delivered as planned.	Attendance of children and parents for health education sessions are assessed by the registry record on the respective session. Fidelity is assessed among the physical education teacher by a questionnaire.

### Step 1: Define the Problem in Behavioral Terms

In this step, researchers are to define the problem in behavioral terms, and there are two components: (i) who would perform the behavior and (ii) what the behavior would be (37). We took into account the specific behavioral context of the problem (i.e., low physical activity levels among Chinese children), and conducted an exploratory literature review to identify the national statistics on physical inactivity and its negative health consequences among Chinese children, and major barriers and facilitators to improving their physical activity (4–7, 48, 49). Specifically, to support our study rationale, an initial search was carried out on MEDLINE and China National Knowledge Infrastructure (CNKI) databases using the keywords: “physical activity,” “physical education,” “Chinese,” “children,” “barriers,” and “facilitators.”

### Step 2: Select the Target Behavior

Having defined the problem of the low level of physical activity in step one, the next step was to decide which behavior to target through the research literature. In this step, a literature search on the trends of physical activity among Chinese children in different physical activity domains (i.e., everyday activity, active recreation, and sport) was undertaken to identify the target behavior that could address the defined problem (as identified in step one) (50–59). Four criteria from the BCW model were used to inform the selection of the final target behavior: (i) how much of an impact changing the behavior would have on the desired outcome, (ii) how likely it is that the behavior can be changed, (iii) how likely it is that the behavior would have a positive or negative impact on other related behaviors, and (iv) how easy it would be to measure the behavior (37).

### Step 3: Specify the Target Behavior

Behavior specification is the step following the selection of the target behavior where the researchers need to specify the behavior in precise and appropriate detail and in its context. As guided by the BCW, six questions were raised in this step to help us specify the target behavior (37). These questions include (i) who would perform the target behavior, (ii) what they would need to do differently to achieve change, (iii) where and (iv) when they needed to do it, (v) how often, and (vi) with whom would they do it. Similar to step one and two, a literature search along with researchers' knowledge of children's physical activity helped us to develop the behavior specification (14, 56, 60–62).

### Step 4: Identify What Needs to Change

We previously conducted a Joanna Briggs Institute (JBI) qualitative systematic review to synthesize the barriers and facilitators to physical activity (as understood, perceived, or experienced) among ethnic Chinese children who reside in the Chinese and non-Chinese territory or among people who had responsibility for them (e.g., ethnic Chinese/non-Chinese parents, teachers) (63, 64). As this intervention is aimed at Chinese children who are residing in China, only barriers and facilitators to physical activity among Chinese children who reside in the Chinese territories were included. Using the TDF domains and definitions as a guide, the identified barriers and facilitators were coded into 14 TDF domains based on similarity in statements and were illustrated with the quotations. The statements and quotations identified from the systematic review were examined to determine (i) any conflicting beliefs within the domain and (ii) the frequency of specific beliefs across the data (i.e., the percent (count) of statements coded for each specific TDF domain out of the total identified statements).

### Step 5: Identify Intervention Functions

Using the mapping table (TDF-intervention function) in the BCW, the intervention functions that most likely affect the behavioral change were selected based on the TDF diagnosis (37). All the intervention functions were then assessed according to their affordability, practicability, effectiveness/cost-effectiveness, acceptability, side-effects/safety, and equity (APEASE) (37).

### Identify Policy Categories (Not Conducted)

As this study aimed to theoretically develop a school-based intervention and was not related to changing policy on physical activity, this step was skipped. However, the evidence accumulated from the development process may provide useful insights related to policy categories (e.g., guidelines, regulations) for achieving behavioral changes.

### Step 6: Identify Behavior Change Techniques and Intervention Contents

BCTs are considered as the “active ingredients” of an intervention to change behaviors (37). Specifically, BCTs are observable, replicable, and an irreducible component of the behavioral change intervention and a postulated active ingredient within the intervention (i.e., the proposed mechanisms of change). Previously, BCTs have been identified in relation to particular types of behaviors (e.g., physical activity), and these behavior-specific “taxonomies” of BCTs have been synthesized. The BCW is linked with a taxonomy of BCTs that allows systematic and transparent selection of the specific BCTs that would best serve the intervention functions (37, 65). As such, this step used BCT taxonomy (BCTTv1) to identify and map the most frequently used BCTs for each intervention function (65). To identify any additional BCTs, a comprehensive matrix developed by Michie and colleagues to map the relevant 59 BCTs from the BCTTv1 to the TDF domains was utilized (37). The intervention content was then identified in the form of BCTs that would help bring about the target behavior. The APEASE criteria were again used to narrow down the most frequently used BCTs for each intervention function (37).

### Step 7: Identify the Mode of Delivery

In the final step of the BCW, it prompts researchers to consider the full range of possible modes of delivering the interventions before deciding the most appropriate one for the particular target behavior and population and setting (37). We identified the modes of delivery for BCTs after taking into consideration the context in which the intervention would be implemented. Specifically, the selection was based on the previous experience of physical activity intervention development in the research team and supplemented by the findings obtained from similar research of physical activity intervention among children (44, 61, 66–71). Once again, the APEASE criteria were used to assess the selection of the modes of delivery (37).

### Step 8: Expert Consultation

A consultation on the intervention was conducted with six academic researchers from the UK (*n* = 4) and China (*n* = 2). Specifically, they have specialized knowledge and professional experience in developing physical activity interventions and/or behavioral change interventions. They were purposively selected to ensure representation of diversity by expertise. Two of the study researchers (HB/KC) have expertise in the field of physical activity and behavior change, and so access to these physical activity and behavior change experts was gained through professional networks within the study team. The intervention materials were shared with them through email, and they reviewed the selection of intervention functions, BCTs, and content as well as the readability, flow of information, and consistency of expressions in the developed intervention. All experts reviewed the intervention materials independently, and their feedback (received *via* email) was used to improve the intervention structure and materials. Specifically, we thoroughly reviewed and discussed each comment given by the experts and then revised the intervention materials accordingly.

### Step 9: Patient and Public Involvement and Engagement in Research

PPIE in research means doing research “with” the patients and public in research (72). In other words, they should be actively involved in research activities as partners and decision-making. In turn, it could make the methods and outcomes more appropriate to research participants (73). In this case, PPIE involvement provided their perspectives, and the aim was not to ensure their “representativeness” (74). We aimed for a variety of perspectives and different viewpoints. Six lay people in China were part of this PPIE. They were the intended user community and purposively selected. Like step eight, we used our lay members' contact to approach these lay people. Specifically, PPIE group included one boy and a girl aged 10–12 years, parents of each child (one father and one mother who had university education and were employed), and two physical education teachers (one male, one female) who had more than 20 years of teaching experience in a local public primary school in China. The intervention materials were shared with them (i.e., children viewed materials for children, parents viewed materials for children and parents, teachers reviewed all materials), and their preferences were identified (i.e., decision was made by considering whom the material was targeting at) and used to finalize the intervention materials (e.g., whether they preferred cartoon or photo for illustrations in the intervention materials and whether they were comfortable with the length of the text in the intervention materials).

### Step 10: Piloting

Piloting is a preparatory investigation that provides specific information needed for planning subsequent studies (75). It can assist the researchers to identify potential problems as well as possible solutions (75). In this step, we tested the participants' understanding of our intervention materials and finalized these. Specifically, we reviewed comments raised about unintelligible, unacceptable, or offensive words within the intervention materials and revisions were made after considering the alternative words or expressions that best represent the acceptable and common language in Chinese culture. The intervention materials were piloted among four children (aged 10–12 years), four parents (whose child was aged 10–12 years), and four physical education teachers. They were purposively selected from a local primary school. Like step nine, we aimed for a variety of perspectives and different viewpoints. The intervention materials were shared with them to read and understand in their free time (i.e., children viewed materials for children, parents viewed materials for children and parents, teachers reviewed all materials). The shared intervention materials were reported in the result section (i.e., step eight, nine, and ten). The objectives were to identify any difficulties they had in reading and understanding the intervention materials (i.e., comprehension of content/instructions). Parents and teachers were requested to make suggestions for change or discuss any queries directly with the researchers. Teachers were requested to collect and record any queries that children had and report these to the researchers. Their feedback was used to finalize the intervention.

## Results

### Step 1: Define the Problem in Behavioral Terms

Physical inactivity among children is a serious public health problem. Evidence suggests that physical inactivity is significantly associated with many negative health (physical and mental) consequences, including increased risk of cardiovascular diseases, cancers, type 2 diabetes, musculoskeletal disorders, anxiety, and depression (48, 49). We identified four systematic reviews that synthesized the reasons for low engagement in physical activity among Chinese children (14, 76–78). In summary, we synthesized that physical inactivity among children was a result of a combination of personal, social-cultural, environmental, and policy- and program-related factors (63, 64). All these factors should be taken into consideration when developing a behavior change intervention to increase physical activity among Chinese children.

### Step 2: Select the Target Behavior

**Figure 2:**
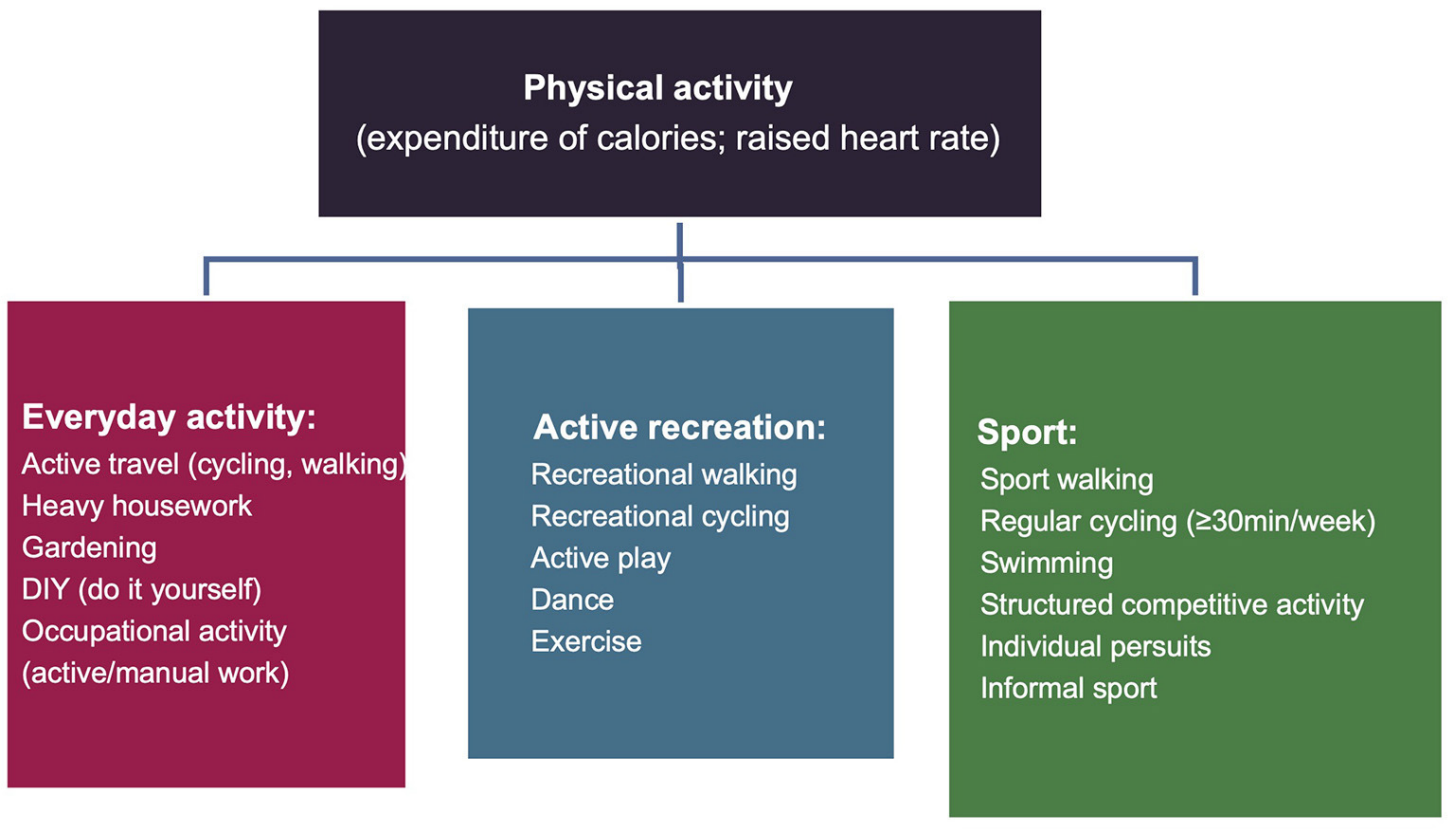
Physical activity domains.

Physical activity is a complex behavior and can be divided into three domains: everyday activity (e.g., active travel, occupational activity), active recreation (e.g., active play, recreational walking, exercise), and sport (e.g., sport walking, structured competitive activity) (see Figure 2) (79). In China, the decline in children's physical activity can be found in all of these three domains, and this decline continues from childhood into adolescence. Regarding everyday activity, for instance, a great decline was reported in children's active travel (50). Specifically, there were only ~41% of Chinese children who traveled to school on foot or by bike in 2016 (51). In terms of active recreation, it has been significantly influenced by the increasing utilization of computer, television (TV), and video games (52). Children should not have more than 120 min of sedentary behavior per day (e.g., watching TV, using computers, playing video games) (53). However, over a third of Chinese children did not achieve this recommendation in 2016 (59). Chinese children are reported to have insufficient physical activity (sport) opportunities, and they spend the majority of their time on academic attainment (54–57). Previous research has reported an average of 9 h in school's weekday (ranges from 6.5 to 11.65 h) in Chinese schools (80). The majority of Chinese children are suggested to spend around 30–90 min per day on homework, and there are 30% of children who spend over 90 min on homework (21, 80). Chinese children have a higher study load than children from other areas of the world (around 14 h per week on homework compared to around 6 h in the US and 3 h in Finland) (80). In addition, more than 66% of Chinese children are having extracurricular tutoring classes each week. In contrast, only 24.7% of Chinese children participated in organized exercise sessions afterschool (6). In China, academic achievements are heavily emphasized and schools are evaluated based on their academic performances. As a result, schools prefer to allocate greater resources (including time) more to academic curriculum compared to physical activity (21, 22, 58). Overall, all these three domains were identified to be the pivotal domains. As suggested by the BCW, it is more effective to intervene intensively on one or two target behaviors than to intervene less intensively on multiple behaviors (37). Children's physical activity through active recreation at school appeared to be promising and could have a positive impact on children and their peers and parents. These criteria addressed that (i) how much of an impact changing the behavior would have on the desired outcome, (ii) how likely it is that the behavior could be changed, (iii) how likely it is that the behavior would have a positive or negative impact on other related behaviors, and (iv) how easy it would be to measure the behavior (37). Children spend the majority time of their waking hours in school, and therefore schools represent an ideal environment to reach the majority of school-aged children (81). Schools can provide access to children from different socioeconomic backgrounds and help institutionalize the physical activity programs into other settings, such as communities (81). Five to 45 min per day of improvement in MVPA can be achieved through school-based physical activity interventions (67). Based on existing literature, targeting schools to increase children's physical activity appears to be promising and is relatively easy to implement and reasonably easy to measure (60–62).

### Step 3: Specify the Target Behavior

The behavior specification is detailed in Table 2. Previous systematic reviews on school-based physical activity interventions have identified that the increase in children's physical activity level can be potentially achieved through (i) active lunch breaks (i.e., providing opportunities for children to be physically active during the lunch break), (ii) classroom physical activity breaks (i.e., short breaks for doing physical activities), and (iii) education on being physically active (60, 61). The classroom physical activity breaks are highly effective in presenting opportunities for children to participate in a range of organized or unorganized physical activities (61, 62). Classroom physical activity breaks were therefore considered as an important intervention target time in our study. In terms of the people who could influence children's physical activity engagement, parents and teachers were identified as the integral “gatekeepers” because they play an important role in establishing children's health behaviors (14, 56). Parents and grandparents are likely to be over-attentive due to the “one-child policy” in China, and this may result in children having less intrinsic physical activity motivations (82). Beyond the parental influences, the role of teachers and peers is influential in shaping a child's physical activity behavior and has been identified as another important facilitator in the literature (14, 56, 76, 77). Altogether, children were identified as the intervention target, and their peers, teachers, and parents were identified as important people who may influence children's physical activities.

**Table 2:** Behavior specification.

(i) Who needs to perform the behavior?	Children (4, 5)
(ii) What do they need to do differently to achieve the desired change?	Participate in more physical activity at school (10, 83)
(iii) When do they need to do it?	Physical education class, break time and after school time (60–62)
(iv) Where do they need to do it?	At school and after school (67, 81)
(v) How often do they need to do it?	At least 60 min MVPA per day (1)
(vi) With whom do they need to do it?	Peers, teachers, parents (14, 56, 76, 77)

### Step 4: Identify What Needs to Change

The findings of the systematic review are reported in detail elsewhere (64). Briefly, 11 studies were included in our systematic review explored the barriers and facilitators to physical activity among ethnic Chinese children and extracted 56 findings. Of which 37 findings were extracted from the studies conducted in the Chinese territories and 19 findings were from the studies in non-Chinese territories. Overall, 10 TDF domains were coded based on the findings of systematic review in this step (see Table 3). Of 37 extracted findings, the most commonly coded TDF domains accounting for 89.1% of the total findings were environmental context and resources (*n* = 12; 32.4%), social influences (*n* = 8; 21.6%), beliefs about consequences (*n* = 6; 16.2%), memory, attention, and decision process (*n* = 3; 8.1%), emotion (*n* = 2; 5.4%), and belief about capabilities (*n* = 2; 5.4%). The remaining findings were coded into other four TDF domains, which accounted for 2.7% (*n* = 1), respectively (e.g., belief about capabilities, skills, knowledge, social/professional role, and identity). Four domains (optimism, reinforcement, intentions, and behavioral regulation) were not identified from the 37 findings and thus were not coded. The table of TDF domains, findings, and quotations was made and organized hierarchically by percent frequency (see Table 3). Of 37 findings, 16 were facilitators and 22 were barriers. Only one finding (circumstances that make physical activity easy and circumstances that make it difficult) was discussed as both barrier and facilitator. In terms of the TDF domains, more barriers than facilitators were discussed relating to environmental context and resources (three facilitators, eight barriers, and one both) while only the social influences had more facilitators than barriers discussed in the domain (five facilitators, two barriers). As for the memory, attention and decision process, emotion, skills, and social/professional role and identity, only the barriers were discussed within these domains. In comparison, only facilitators were discussed in the domains of belief about capabilities, knowledge and motivation, and goals. Barriers and facilitators were equally discussed in beliefs about consequences.

**Table 3:** Summary of behavioral diagnosis using the TDF (quotes are taken verbatim from the systematic review).

**TDF**	Frequency^*^ (% total)	**Findings (barriers and facilitators)/why are children doing or not doing physical activity**	**Quotes**
Environmental context and resources	32.4%	I have no time or peers to play with me for leisure-time exercise (B)	“I do not have time for leisure time exercise at all from Monday to Friday. I came home late from school in the evening, and when I finish my homework, it's time to sleep. I just don't have time to exercise at all”
		Salient consequences of participating in physical activity (B)	“The most frequently mentioned disadvantage, “will take too much time,” was mentioned by 40.6% of the students”
		Circumstances that make physical activity easy and circumstances that make it difficult (B, F)	“Many of the circumstances (e.g., assignments, time, and weather) were mentioned both as circumstances that make physical activity easy and as circumstances that make it difficult. The most frequently reported facilitator of physical activity, “having fewer assignments,” was mentioned by 27.7% of the students. The most frequently mentioned barrier, “having too many assignments,” was mentioned by about half (48.4%) of the students. Additionally, “time” was the second most frequently mentioned circumstance; 14.2% of the students mentioned having more time as a facilitator, and 18.8% mentioned not having enough time as a barrier. These data also suggest that “having fun activities,” “having others to participate with,” “approval from others” and “making facilities more available” operate as facilitating and hindering circumstances”
		“Academic-focused” school environment seemed to be a major barrier for PA participation (B)	“I personally think childhood obesity nowadays is due to the fact that kids eat too much and move too little. The environment now is so different from the environment of my childhood. My kid is sitting there all day studying and no time for exercise. It seems that he does not like exercise at all, and when he has spare time, he watches TV”
		Safety (crime) (B)	“Afraid of being taken or hurt at night”
		Safety (traffic) (B)	“Few cars on roads”
		Safety issue (B)	“Some dangerous activities, like skating, it's not too safe for my girl to play. Children usually can't decide what is dangerous for them. They just play for fun but neglect the importance of safety. For example, I won't let him go climbing hills or rock…it's too dangerous. I've seen quite a lot of accidents happened in people climbing, it's definitely not suitable for children, especially girls, it's just not right for her to play such rough sport and Hong Kong don't have much safe climbing places for younger ones and there is a lack of qualified teachers”
		Functionality (F)	“Convenient transportation”
		Other negative feelings (B)	“Too many students in a class (40–45 students sharing one basketball court)”
		Destination (F)	“Recreation grounds”
		Aesthetic (F)	“Fresh air”
		Others (B)	“Too many people in recreation grounds”
Social influences	21.6%	Families are an important factor in their physical activity participation (F)	“Doing physical activity is fun and when I see other people playing, I want to be one of them … My parents also encouraged me. They would spend time jogging with me and I still remember we had so much fun”
		Confucian father played a leading role in determining the different dimensions in his children's life (F)	“Interestingly, within the group we interviewed, only fathers provided actual facilitation of their children's physical activity. One of them acted as an assistant coach regularly in his boy's rugby team, while a few attended their children's sports competitions such as badminton and swimming during their leisure time, and two parents assisted with transporting children to playgrounds”
		Salient who approve referents (F)	“Clearly, most of the salient referents for this behavior were family members, including parents, others, fathers, and grandparents. Teachers and classmates were also mentioned by these middle school students. Doctors and other health professionals were not very frequently mentioned. Parents were the most frequently mentioned approving group and disapproving group and were mentioned more frequently as approving (42.6%) than as disapproving (27.7%)”
		High expectation of children's academic success (B)	“My son is now in the soccer team and he practices for many days a week … he comes back home at 7 pm and I think soccer is distracting him from studying well … I'm so afraid that he'll get hurt and I'm thinking not let him play anymore. I usually let him do whatever he wants in sports but if the exam is near, I'd advice him to play less basketball, I think it's better for him to spend more time studying than on playing”
		Physical activity to be another responsibility in their lives (B)	“There was a time a basketball club invited me to join them, and I know this was a chance for me to play at a professional level. However, my mother opposed my decision, and I did not dare to argue with her. Well, actually I don't really feel too regretful as I treated it as an interest. It only matters if I could play basketball happily or not. After practicing for a long time, I saw there was not much improvement, and I felt that I had reached the top already. Therefore, I gave up the interest in swimming”
		Make tailored objectives for students (F)	“We should combine the collective goal with the individual goal. Each student's physical quality and individual ability are different. We should fully consider the individual difference of each student when setting the teaching goal. When setting the collective teaching goal, we should make the goal has a certain range of fluctuation, because the requirement is universal for each student. We should ensure that students whose sports learning ability are not strong enough can improve their sports achievements through hard work and their interest in sports learning can be increased due to the establishment of motivation in sports learning. In the other way, this goal should also apply to those who have higher sports learning abilities”
		Strength the teaching and management regulation (F)	“The PE teachers should respect and care about students rather than criticize students at will. Teachers should equip a positive attitude and be the role model so that the students will feel the equality between themselves and teachers. In addition, they may be attracted to the class. With the establishment of a harmonious relationship between students and teachers, students will thus take the teachers as examples and change their behavior”
		Lack of social support (B)	“I always do sport alone by myself and this is why sport is not interesting to me. I do not like it because there is no one to compete with or compare with me. There is no one to encourage me and I do not feel happy when doing sport”
Beliefs about consequences	16.2%	Improving on their health (F)	“Physical activity is important because it is good for my health, I get less pain and disease after doing physical activities”
		Having positive outcomes for academic and/or career future (F)	“Sport may be useful for my future job because I will find sport-related careers, like being an athlete. I want to be as good as those Olympic athletes, I think they are gorgeous”
		Physical activity was not useful in relation to entering a better secondary school (B)	“Sometimes I think it [physical activity] is not really that useful at all as most schools do not think it is relevant to study”
		Physical activity had a lower priority than academic studies (B)	“I would like to excel in my academic studies. I think studies may affect my future but not the sport. I always place academic studies first”
		The importance of PE was linked closely to their children's academic advancement (F)	“PE is important because it makes my son healthier … good health may help him study better”
		Confucian beliefs in taking ‘good care’ of children (B)	“I used to do some sports and stretching exercises at home. I don't participate in too many activities now after I've had my child. I need to take care of her academic matters and other aspects of life such as driving her to a private tutoring center to learn English and taking a computer course after school. Therefore, I don't have much time to do exercise myself. I know doing more physical activity is good for my child, but I'd rather have her study first and only allow her to play for a while if she could finish her school work. You know, too much play will negatively affect her academic performance”
Memory, attention and decision process	8.1%	Feeling too tired after doing physical activity (B)	“I need to put in too much effort in sport. I do not like the feeling of tiredness after doing sport”
		I exercise only during the physical education (PE) class, and I exercise primarily to pass the high school entrance examination, commonly known as “Zhongkao.” (B)	“We have a morning recess. Usually, we start with group rhythmic gymnastics and then jump roping. We have PE class, and each class content is arranged by teachers for us to run or do items for Zhongkao. We are not given free play time during PE class. I like playing badminton, but my PE teacher said I can only play badminton after I am capable of receiving full credits for all Zhongkao-tested items”
		Complained about the teacher and the curriculum (B)	“Having academic subject lessons at the expense of PE lessons, especially when it comes to the senior secondary level”
Emotion	5.4%	Overly skill-oriented nature of their classes (B)	“It's not very nice because we always learn traditional sports, such as track and field, soccer, basketball, volleyball, and gymnastics. It is no fun at all. Teachers are actually repeating the same content we have already learned in senior primary schools. I dislike running long distances; it is so boring”
		Insufficient time provision both in the school's physical education curriculum and after school hours (B)	“Well, it seems that during PE lessons, most of the time, about 15–20 min was allocated to doing stretching and warm-up, and the time for us to really learn a sport is not enough”
Belief about capabilities	5.4%	Feeling happy and competent and enjoying the sport and movement (F)	“I feel satisfied when I can shoot a basketball into the ring … and usually, I am able to hit the shuttlecock with a swift sound and therefore I feel really happy and have fun playing with it … When I play badminton, I think the sound of hitting a badminton cock is really interesting, I feel very happy when I could hit the cock with that kind of special sound”
		High attainment value and high utility in physical activity (F)	“I can boast in front of my teammates in basketball, which I think is important for me to do better in it”
Goals	2.7%	As they grew up, time spent on a particular physical activity increased (F)	“I play table tennis more now and therefore have less time for other activities. I'd rather focus my time on improving in it than spending time doing various sports which seem to be wasting my time”
Skills	2.7%	Lack of perceived improvement in physical activity (B)	“I do not feel any differences or improvements and I think it is boring. I am always like that, not a bit better”
Knowledge	2.7%	An instrumental orientation to physical activity engagement (F)	“Students in Hong Kong, as far as I know, are not doing much sports and exercise. I'm sure exercise can help my son to be fit and maintain a good shape and weight”
Social/professional role and identity	2.7%	Parental work commitment (B)	“Both of us need to work 6 days a week, well… we're not rich and we must work for the money for the family. Sometimes we would go out with the kids on Sundays and we usually go shopping or dining out”

*B, barrier; F, facilitator*.

* *%, the percent (count) of statements coded to each specific TDF domain, out of the total identified statements*.

### Step 5: Identify Intervention Functions

All nine intervention functions were available for selection. Two intervention functions (i.e., coercion and restriction) were excluded as they did not meet the APEASE criteria (37). Coercion was not considered as practicable, acceptable, or equitable in the school context. The restriction was deemed not to be acceptable to children, parents, and teachers. Eventually, seven intervention functions were selected for this study design including education, persuasion, incentivization, training, environmental restructuring, modeling, and enablement.

### Step 6: Identify Behavior Change Techniques and Intervention Contents

In terms of the BCTs, 15 BCTs that best serve the seven intervention functions were first identified using the BCTTv1 taxonomy and then selected based on evaluation against the APEASE criteria (37, 65). Subsequently, a review of the matrix of TDF and BCTs identified six additional BCTs to include in this intervention (see Table 4) (84). As a result, there were 21 BCTs in total selected for the intervention.

**Table 4:** Selected BCTs for this intervention.

**TDF**	**Intervention function**	**BCT**	**Does the BCT meet the APEASE criteria?**
Skills	Training	Demonstration of the behavior	No: not acceptable
		Instruction on how to perform a behavior	No: not acceptable
		Feedback on the behavior	Yes: provide feedback on the behavioral performance
		Feedback on outcome(s) of behavior	No: not practicable or acceptable
		Self-monitoring of the behavior	Yes: incorporate a measuring method for own physical activity level
		Behavioral rehearsal/practice	No: not practicable, effective, or acceptable
		**Graded tasks**	Yes: include easy-to-perform tasks
Knowledge	Education	Information about social and environmental consequences	No: not effective or acceptable
		Information about health consequences	Yes: educate on the health outcomes of physical activity
		Information on emotional benefits	Yes: educate on the emotional outcomes of physical activity
		Feedback on the behavior	No: not effective or acceptable
		Feedback on outcome(s) of the behavior	No: not effective or acceptable
		Prompts/cues	No: not practicable or acceptable
		Self-monitoring of the behavior	No: not effective or acceptable
Memory, attention and decision processes	Training	Demonstration of the behavior	Yes: provide observable examples of children who are enjoying the physical activity
		Instruction on how to perform a behavior	Yes: give advice about how to perform the behavior
		Feedback on the behavior	No: not practicable, effective, or acceptable
		Feedback on outcome(s) of behavior	No: not practicable, effective, or acceptable
		Self-monitoring of the behavior	No: not practicable, effective, or acceptable
		Behavioral rehearsal/practice	No: not practicable, effective, or acceptable
	Enablement	Social support (practical)	Yes: advice, arrange or provide practical help to do physical activity
		Goal setting (behavior)	No: not practicable, effective, or acceptable
		Goal setting (outcome)	No: not practicable, effective, or acceptable
		Adding objects to the environment	No: not practicable, effective, or acceptable
		Problem-solving	No: not practicable, effective, or acceptable
		Action planning	No: not practicable, effective, or acceptable
		Self-monitoring of the behavior	No: not practicable, effective, or acceptable
		Restructuring the physical environment	Yes: advice changing the “exam-oriented” physical education environment
		Review behavior goal(s)	No: not practicable, effective, or acceptable
		Review outcome goal(s)	No: not practicable, effective, or acceptable
Social/professional role and identity	Education	Information about social and environmental consequences	No: not practicable or acceptable
		Information about health consequences	Yes: educate on the outcomes of physical inactivity
		Information on emotional benefits	Yes: educate on the emotional outcomes of physical activity
		Feedback on the behavior	No: not practicable or acceptable
		Feedback on outcome(s) of the behavior	No: not practicable or acceptable
		Prompts/cues	No: not practicable or acceptable
		Self-monitoring of the behavior	No: not practicable or acceptable
	Persuasion	Credible source	Yes: present verbal or visual communication from a credible source against the physical inactivity
		Information about social and environmental consequences	No: not practicable or acceptable
		Information about health consequences	Yes: persuasive communication on the benefits of exercises for children
		Feedback on behavior	No: not practicable or acceptable
		Feedback on outcome(s) of the behavior	No: not practicable or acceptable
	Modeling	Demonstration of the behavior	Yes: provide observable examples where parents play with their child
Belief about capabilities	Education	Information about social and environmental consequences	No: not practicable or acceptable
		Information about health consequences	Yes: educate on the outcomes of physical inactivity
		Feedback on the behavior	Yes: provide informative on the behavioral performance
		Feedback on outcome(s) of the behavior	No: not practicable or acceptable
		Prompts/cues	No: not practicable or acceptable
		Self-monitoring of the behavior	Yes: incorporate a measuring method for own physical activity level
		**Focus on past success**	Yes: advice to think about or list previous successes in doing physical activity
		**Verbal persuasion about capability (to boost self-efficacy)**	Yes: tell the children that they can successfully do any (paly) physical activity even they may not good at it now
	Persuasion	Credible source	No: not effective or acceptable
		Information about social and environmental consequences	No: not effective or acceptable
		Information about health consequences	No: not effective or acceptable
		Feedback on behavior	Yes: provide feedback on the behavioral performance
		Feedback on outcome(s) of the behavior	No: not practicable or acceptable
	Modeling	Demonstration of the behavior	Yes: provide observable examples of children/sportsman who have successfully participated in sports/exercises
Belief about consequences	Education	Information about social and environmental consequences	No: not effective or acceptable
		Information about health consequences	Yes: educate on the outcomes of physical inactivity and benefits of physical activity
		Information on emotional benefits	Yes: educate on the emotional outcomes of physical activity
		Feedback on the behavior	No: not practicable, effective, or acceptable
		Feedback on outcome(s) of the behavior	No: not practicable, effective, or acceptable
		Prompts/cues	No: not practicable, effective, or acceptable
		Self-monitoring of the behavior	No: not effective or acceptable
		**Pros and cons**	Yes: educate on the outcomes of physical inactivity and benefits of physical activity
	Persuasion	Credible source	No: not practicable or acceptable
		Information about social and environmental consequences	No: not effective or acceptable
		Information about health consequences	Yes: educate on the outcomes of physical inactivity and benefits of physical activity
		Feedback on behavior	No: not practicable, effective, or acceptable
		Feedback on outcome(s) of the behavior	No: not practicable, effective, or acceptable
	Modeling	Demonstration of the behavior	Yes: provide observable examples of benefits obtained by the children from doing sports/exercises
Goals	Education	Information about social and environmental consequences	No: not practicable, effective, or acceptable
		Information about health consequences	No: not practicable, effective, or acceptable
		Feedback on the behavior	Yes: provide feedback on the physical activity
		Feedback on outcome(s) of the behavior	No: not practicable, effective, or acceptable
		Prompts/cues	Yes: have physical activity poster posted in the classroom
		Self-monitoring of the behavior	Yes: incorporate a measuring method for own physical activity level (e.g., pedometer diary)
	Persuasion	Credible source	No: not practicable, effective, or acceptable
		Information about social and environmental consequences	No: not practicable, effective, or acceptable
		Information about health consequences	No: not practicable, effective, or acceptable
		Feedback on behavior	Yes: provide feedback on the physical activity
		Feedback on outcome(s) of the behavior	No: not practicable, effective, or acceptable
	Incentivization	Feedback on behavior	Yes: provide feedback on the physical activity
		Feedback on outcome(s) of behavior	No: not practicable, effective, or acceptable
		Monitoring of behavior by others without evidence of feedback	No: not practicable, or acceptable
		Monitoring outcome of behavior by others without evidence of feedback	No: not practicable, acceptable, or effective
		Self-monitoring of the behavior	Yes: incorporate a measuring method for own physical activity level (e.g., pedometer diary)
		Material reward	Yes: provide stickers for children who have achieved physical activity goals
	Modeling	Demonstration of the behavior	No: not practicable, effective, or acceptable
	Enablement	Social support (practical)	Yes: advice parents to do physical activity with children
		Goal setting (behavior)	No: not practicable, effective, or acceptable
		Goal setting (outcome)	No: not practicable, effective, or acceptable
		Adding objects to the environment	No: not practicable, effective, or acceptable
		Problem-solving	No: not practicable, effective, or acceptable
		Action planning	No: not practicable, effective, or acceptable
		Self-monitoring of the behavior	No: not practicable, effective, or acceptable
		Restructuring the physical environment	Yes: advice changing the structure of physical education
		Review behavior goal(s)	No: not practicable, effective, or acceptable
		Review outcome goal(s)	No: not practicable, effective, or acceptable
		Social support (emotional)	Yes: advice parents and teachers to provide positive feedback/encouragement
Emotions	Enablement	Social support (practical)	Yes: advice, arrange or offer practical help to do physical activity
		Goal setting (behavior)	Yes: advice children to make physical activity plans
		Goal setting (outcome)	No: not practicable or acceptable
		Adding objects to the environment	No: not practicable, effective or acceptable
		Problem-solving	Yes: discussion on barriers and provision of feedback
		Action planning	No: not practicable, effective, or acceptable
		Self-monitoring of the behavior	No: not practicable, effective or acceptable
		Restructuring the physical environment	Yes: advice changing the “exam-oriented” physical education's environment
		Review behavior goal(s)	No: not practicable, effective, or acceptable
		Review outcome goal(s)	No: not practicable, effective, or acceptable
		**Social support (emotional)**	Yes: provide emotional encouragement and positive feedback
Environment context and resources	Environmental restructuring	Adding objects to the environment	Yes: provide sports equipment for children
		Prompts/cues	Yes: have physical activity poster posted in the classroom
		Restructuring the physical environment	No: not affordable, practicable, or acceptable
	Enablement	Social support (practical)	Yes: advice parents/teachers to encourage or do physical activity with children
		Goal setting (behavior)	Yes: agree on a daily walking goal with children
		Goal setting (outcome)	No: not practicable or acceptable
		Adding objects to the environment	Yes: provide sports equipment for children
		Problem-solving	Yes: prompt the children to identify barriers that prevent them from doing physical activity and discuss ways in which they could overcome the barriers
		Action planning	Yes: encourage to make a plan to do physical activity on weekends
		Self-monitoring of the behavior	Yes: give the child a pedometer and a diary for recording the daily total number of steps
		Restructuring the physical environment	Yes: provide sports equipment for children
		Review behavior goal(s)	Yes: examine how well a person's performance corresponds to agreed goals
		Review outcome goal(s)	No: not practicable or acceptable
Social influences	Modeling	Demonstration of the behavior	Yes: provide observable examples of parents who encourage children to do physical activity or play with their child
	Enablement	Social support (practical)	Yes: advice parents/teachers to encourage or do physical activity with children
		Goal setting (behavior)	Yes: agree on a daily walking goal with children
		Goal setting (outcome)	No: not practicable or acceptable
		Adding objects to the environment	Yes: provide sports equipment for children
		Problem-solving	Yes: prompt the children to identify barriers that prevent them from doing physical activity and discuss ways in which they could overcome the barriers
		Action planning	Yes: encourage to make a plan to do physical activity on weekends
		Self-monitoring of the behavior	Yes: give the child a pedometer and a diary for recording the daily total number of steps
		Restructuring the physical environment	Yes: provide sports equipment for children
		Review behavior goal(s)	Yes: examine how well a person's performance corresponds to agreed goals
		Review outcome goal(s)	No: not practicable or acceptable
		**Social support (emotional)**	Yes: advice parents and teachers to give positive feedback/encouragement
		Identification of self as role model	No: not practicable or acceptable
		**Social reward**	Yes: verbally praise the children who have achieved the goal

*Embolden BCTs were identified through the TDF-BCT matrix*.

A summary of intervention functions, BCTs as well as examples of intervention contents that describe how the BCTs are delivered in the intervention is presented in Table 5. This table represents a summary of all the steps in the BCW and is the culmination of all the steps of the work in the current study. As suggested in step four, the barriers and facilitators were, by and large, related to the TDF domains of environmental context and resources; social influences; the belief about the consequences; memory, attention and decision process; and emotion. In other words, environmental, socio-cultural, and personal factors would be the priorities of our intervention, and the selected BCTs would help bring about the target behavior. Restructuring the physical environment is to make a change in the physical environment to help facilitate the performance of the target behavior (37). By adding objects to the environment (providing sports equipment) and using prompts/cues (e.g., poster), children's initiatives for doing physical activity may increase (see Table 5). The teachers will persuade the role of physical activity and encourage children to use the provided sports equipment.

**Table 5:** Examples of the intervention content and mechanism of action using the BCW and TDF.

**Intervention contents (examples of application within the intervention)**	**BCTs selected**	**Intervention functions**	**TDF**	**COM-B**
**School environmental support**				
Verbal encouragement for doing physical activity during the class break.	15.1 Verbal persuasion about capability	Pers	B cap	Ref M
Put physical activity posters in the classroom.	7.1 Prompt/cues 12.5 Adding objects to the environment	Ed, Env	Env, Goals	Ref M, Phy O
The intervention will provide sports equipment for children to use during the class break.	3.2 Social support (practical) 12.1 Restructuring the physical environment 12.5 Adding objects to the environment	Ena, Env	Env, MAD	Phy O, Psy C
**Health education for children**				
General introduction to physical activity *via* slides (definition, positives, negatives of physical inactivity).	5.1 Information about health consequences 5.6 Information on emotional benefits	Ed	B con, Kn	Ref M, Psy C
Discussion about what sort of things make them want to do physical activity, what stops them from doing it, and the previous successful physical activity experience.	1.2 Problem-solving 9.2 Pros and cons 15.3 Focus on past success	Ed	B cap, Kn	Ref M, Psy C
Tips for doing physical activity (intense, duration, safety) and motivation.	4.1 Instruction on how to perform the behavior 6.1 Demonstration of the behavior	Pers	B cap	Ref M
Encourage children to make physical activity plans to be active at school and home gradually. Whenever the goal or plan is achieved, verbal congratulations or non-verbal rewards will be offered to them.	1.1 Goal setting (behavior) 1.4 Action planning 10.2 Material reward (behavior) 10.4 Social reward 15.1 Verbal persuasion about capability	Pers, Inc	B cap	Ref M
Encourage children to make physical activity poster and give the presentation.	1.1 Goal setting (behavior) 1.4 Action planning	Pers, Ena	B cap, B con	Ref M
Provide follow-up educational material (e.g., activity diary) for each child.	5.1 Information about health consequences 5.6 Information on emotional benefits 7.1 Prompts/cues 12.1 Restructuring the physical environment 12.5 Adding objects to the environment	Ed, Env	B con, Kn, B cap, SI	Soc O, Ref M, Psy C
Children will be asked to set up activity goals and record their daily physical activity.	1.1 Goal setting (behavior) 1.4 Action planning 1.5 Review behavior (goals) 2.3 Self-monitoring of the behavior 8.7 Graded tasks	Ena, Tra	Sk, Goals	Phy C, Ref M
Provide feedback with children about the activity plans.	2.2 Feedback on behavior	Pers	B cap	Ref M
**Family involvement**				
Educate parents about the physical activity knowledge and ways to promote physical activity among children.	5.1 Information about health consequences 5.6 Information on emotional benefits	Ed, Pers	Kn, Id	Psy C, Ref M
Use the video clips as the demonstrations (from experts).	6.1 Demonstration of the behavior	Ed, Mod	B con, Kn	Ref M, Psy C
Encourage parents to discuss the barriers that hinder children's physical activity as well as the facilitators that can enable physical activity among children.	1.2 Problem-solving 9.2 Pros and cons	Ed	B cap, Kn	Ref M, Psy C
Encourage parents to support children in doing physical activity (e.g., making action plans).	1.1 Goal setting (behavior) 3.2 Social support (practical) 3.3 Social support (emotional)	Ena	SI, Em	Soc O, Auto M
Provide follow-up educational material (e.g., physical activity booklet) for each parent to reflect on the knowledge of physical activity.	4.1 Instruction on how to perform the behavior 5.1 Information about health consequences 5.6 Information on emotional benefits 7.1 Prompts/cues 12.1 Restructuring the physical environment 12.5 Adding objects to the environment	Ed, Env	SI, Kn, Id, B con, B cap	Soc O, Psy C, Ref M

*TDF domain abbreviations: Kn, knowledge; MAD, memory, attention and decision processes; SI, social influences; Env, environmental context and resources; B Cap, beliefs about capabilities; B Con, beliefs about consequences; Id, social/professional role and identity; Em, emotions; Sk, physical skills. COM-B abbreviations: Psy C, psychological capability; Soc O, social opportunity; Phys O, physical opportunity; Ref M, reflective motivation; Auto M, automatic motivation. Intervention function abbreviations: Ed, education; Pers, persuasion; Env r, environmental restricting; Mod, modeling; Ena, enablement; Tra, training; Inc, incentivization*.

Provision of information about health consequences/emotional benefits in the intervention could educate both children and parents. Additionally, the demonstration of the behavior could act as persuasion whenever there exists an opportunity for doing physical activity (see Table 5). Emphasizing the pros and cons, previous successful experience, and personal capacities in physical activity may encourage children to perform the activity voluntarily. Information that indicates when and how to perform physical activity is crucial content for the individuals and could act as a prompt or cue to action (see Table 5). Goal setting, graded tasks, and action planning were also identified as valuable BCTs and emphasize the importance of acting in the present but also planning for the future. Engaging children to self-monitor the behavior may facilitate the completion of individual actions and act as a springboard from which children can improve with feedback (on their behavior) from a health practitioner. Moreover, teachers and health practitioners will review the behavioral goals of children through the intervention to encourage them to act on their own self-set personalized goals (see Table 5). Other social support strategies will involve asking children to buddy up with friends [i.e., social support (emotional)] or parents to prepare sports gear for their children [i.e., social support (practical)]. Whenever a child achieves the physical activity goal, giving a social reward or material reward may help incentivize their motivation in keeping the desired behavior.

### Step 7: Identify the Mode of Delivery

Several systematic reviews suggest that school-based interventions are the most effective way to counteract low physical activity in children aged 5–18 years, and it is typically made up of a combination of school curricula, printed educational materials, educational sessions, and physical activity-specific sessions (66, 67). In addition, previous studies have demonstrated the effectiveness of group-based sessions and printed media (e.g., poster, written materials) in helping increase children's physical activity level (44, 61, 68–71). As a result, the face-to-face (group) and distance (printed media) were selected as the modes of delivery.

### Step 8, 9, 10: Expert Consultation, PPIE, and Piloting

As part of the intervention, we adapted a physical activity booklet, poster, slides, and two physical activity diaries (see Table 5). With permission, the information on children's physical activity, reasons behind the physical inactivity among children, and recommendations for helping increase children's physical activity were extracted from two existing booklets of the British Heart Foundation (UK), the websites of Better Health Channel (Australia), Caring for Kids (Canada), and two WHO reports (1, 85–89). These were adapted to the Chinese context and used in the physical activity booklet and slides for parents. The physical activity booklet was distributed among teachers to use as guidance for health education practices. In addition, the intervention includes video clips (i.e., physical activity expert's speech) to provide an introduction about children's physical activity among parents. The content of the slides for children was based on existing slides of the Zhejiang Center of Disease Control (China), and the poster was adapted from a poster produced by the US Center for Disease Control and Prevention (90, 91).

Rather than developing a completely new dairy, we adapted two existing theory- and evidence-based physical activity diaries (Steps for Active Kids (STAK) and STAK-Diabetes, UK). Both diaries were found to be effective in engaging children in physical activities (70, 71). With permission, the information on physical activity knowledge, recommendations to become physically activity and activity log to record daily physical activity, was extracted from both diaries as well as the Better Health Channel's website (70, 71, 87), and the materials were then adapted to the Chinese context. Two BCTs (information on emotional benefits and material rewards) were added to the intervention based on expert consultation. In addition, the disagreements that arose regarding the selection of certain intervention functions and BCTs (i.e., not appropriate or wrong coding) were addressed through iterative revisions until the consensus was reached. The intervention content, complex language, lengthy wording, inconsistent use of phrases and color scheme, and typos were addressed based on the expert consultation. For instance, experts' comments regarding the intervention content and complex language included:

- “*Promoting a health behavior like physical activity does not mean that you must promote the health benefits of that behavior. Instead, you should focus on encouraging people to pursue activity for some intrinsically valued motive*.”
- “*Have you considered the whole issue of financial constraints and how to deal with it. For example, parents might say the only thing their daughter really wants to do is horse riding but they cannot afford it*.”
- “*The language used throughout the resources is quite technical/advanced*.”
- “*…particularly the slides both for parents/children and the booklet are that they are far too complex (especially for young children) and there is a lot of scientific language that is not appropriate for members of the public/children*.”
- “*The participant-facing wording is technical and not lay-friendly*.”
- “*Consider using ‘mental health’ instead of ‘psychological’*.”

Experts' comments regarding the length wording included:

- “*I have included suggestions of ways you could think about simplifying some of the language, particularly in the slides for the children in the comments function on PowerPoint, … see where you can simplify the language and cut down on as much text as possible where you can*.”
- “*You should simplify your wording throughout, so that it speaks to participants in their own language; not necessarily informal language, but a more simple and direct form of wording that resonates with them*.”

Experts' comments regarding the inconsistent use of phrases, color scheme, and typos included:

- “*I think you should use the language from the activity diary in your slides/booklet and use that consistently across all the intervention materials*.”
- “*The color scheme throughout these resources could be more consistent*.”
- “*Keep font size consistent throughout. Including size of titles*.”

The cartoons (for physical activity illustration) used in the activity diary and slides for children were chosen based on the feedback received from children. The photo (for physical activity illustration) used in the physical activity booklet and slides for parents were chosen based on the feedback received from parents and teachers. For instance, PPIE comments regarding the preference of the photograph and length wording included:

- “*I would prefer the cartoons because these are more appealing.”*
- “*I think cartoons are interesting and our teachers make slides in this way [use cartoons as illustrations] as well.”*
- “*I think cartoons are too childish for us [parents] and I would like the photo instead.”*
- “*I would suggest you use photos as illustrations in the materials for parents.”*
- “*I think the length is fine.”*
- “*I am fine with the length.”*

All these intervention materials are available in English and Mandarin. The translation work was completed after PPIE. Translational and literal mistakes in the Mandarin version were corrected after the piloting work. For instance, comments from piloting regarding the translational (including unintelligible expressions), literal mistakes, and recommendations included:

- “*The translation of title ‘Welcome to physical activity promotion’ program (not clear).”*
- “*Translation of ‘Tips for safely doing physical activity’ (not clear).”*
- “*Suggest to re-translate the phrase ‘become more active.”*
- “*Translation - Be creative and vary your child's activities (unclear).”*
- “*Please give the definition and benefits of physical activity in the end of presentation rather than give a brief summary. This could make children have a direct understanding and it may be easier for them to memorize.”*

## Discussion

We report the systematic development of a physical activity program for Chinese children aged 10–12 years. Physical activity interventions for Chinese children have previously been criticized for being underdeveloped or underreported (28–32). Among health practitioners and researchers, it is argued that a systematic and transparent theory-and-evidence-based approach can prevent research waste that is led by poor-quality research methods or researchers' biased manner. In our study, we supplemented this process with findings from a JBI qualitative systematic review (63, 64). This strengthens the likelihood of generalization as the intervention is based on a broader perspective that includes different contexts (e.g., school, home, community) and participants (children, parents, teachers). To our knowledge, the current study is the first to apply the BCW and TDF in this population and context. This systematic process identified 10 relevant TDF domains and selected seven interventions and 21 BCTs in the program. Three main components constitute the intervention, namely: (i) school environmental support, (ii) health education for children, and (iii) family involvement. The school environmental support is based on the provision of sports equipment, pedometers, and a physical activity poster. Health education for children is delivered *via* face-to-face mode using presentation slides and print materials including a physical activity diary. Family involvement is promoted *via* a face-to-face workshop and a physical activity booklet. Moving forward, a feasibility pre- and post-intervention study is being conducted to determine the feasibility of undertaking a cluster randomized controlled trial (RCT) among Chinese children. The feasibility study has been registered with the Chinese Clinical Trial Registry (ChiCTR1900026865). If the intervention is found to be feasible and acceptable, we will design and conduct the cluster RCT.

In the cluster RCT, if the intervention is found to be effective, it could be a low-cost, acceptable and local solution for increasing the physical activity level among Chinese children. It may also alleviate the future personal and economic burden of physical inactivity on individuals, the health system, and the economy. The advantages of increasing physical activity in this population may extend to the prevention of physical inactivity related complications. This program of work means that evidence-based practices could be available to health professionals to promote physical activity among children. The intervention may simultaneously empower children or their parents to actively engage in physical activity. Given that physical inactivity and its related costs are global concerns, there could be worldwide interest in this low-cost behavior change theory-based physical activity program.

## Strengths and Limitations

This systematic approach provided insights to inform the development of a school-based physical activity program for Chinese children. We used the MRC framework along with existing evidence, which led to a logical, practical, and theory-based intervention. The use of the BCW and TDF helped categorize and comprehend potential barriers and facilitators to Chinese children's physical activity. Although several subjective and pragmatic decisions were made throughout the development process, detailed and transparent processes are presented in this paper to clarify why options were or were not taken. We conducted expert consultation on the intervention design, and this minimized risk of bias in the identification of domains, intervention functions, BCTs, and increased trustworthiness. In addition, the PPIE ensured the intervention would be appropriate in the Chinese context. Altogether, the developed intervention will be followed by a thorough evaluation (feasibility pre- and post-intervention study).

This work has several limitations. We identified our target behavior problem (in step 1) through an exploratory literature review and not a systematic review as there are existing systematic reviews and other primary studies to support this. The total count (frequency) of barriers or facilitators coded to each TDF domain was used as a proxy for importance. However, domains that coded infrequently might be highly important to determine the physical activity level among children. Therefore, the design and selection bias may delimit the effectiveness of the intervention. However, it should be noted that using the integrative theoretical framework (i.e., TDF) could enable all the necessary elements for our intervention program are in place to maximize potential benefits. As we were not primarily concerned with changing the policy in this study, the analysis of policy categories was not taken. Further studies are needed to carry out the analysis related to policy categories to help identify guidelines, regulations, and legislations useful for achieving behavioral change. Although expert consultation, PPIE, and piloting were part of the intervention development process, there could be selection bias as convenience sampling was used throughout, and recruitment for this input was undertaken through the researchers' professional and social networks. BCW provides a systematic, theory-driven, and evidence-based approach to develop an intervention that meets the unique needs of the target group. However, the process was time-consuming from problem identification to intervention design.

## Conclusion

This paper describes how an intervention to increase physical activity in Chinese children was developed. We followed a systematic and transparent process using the BCW and TDF to exemplify the first phase (i.e., intervention development) when developing and evaluating a complex intervention. A school-based intervention facilitated by environmental support, health education, and family involvement may help to engage children in physical activity. A feasibility study is now being undertaken in the next phase to determine the acceptability and utility (initial estimates) of the program and the feasibility of undertaking a cluster RCT.

## Data Availability Statement

The original contributions presented in the study are included in the article/supplementary material, further inquiries can be directed to the corresponding author.

## Ethics Statement

The study involving human participants was reviewed and approved by the Faculty of Medicine & Health Sciences Research Ethics Committee, University of Nottingham.

## Author Contributions

HW took the lead in writing the manuscript. HB and KC supervised the project. All authors provided critical feedback and helped shape the research, analysis, and manuscript.

## Conflict of Interest

The authors declare that the research was conducted in the absence of any commercial or financial relationships that could be construed as a potential conflict of interest.
